# The Role of PAK1 in the Maturation of Invadopodia During Transient Mechanical Stimulation

**DOI:** 10.3389/fcell.2019.00269

**Published:** 2019-11-06

**Authors:** Alexander N. Gasparski, Jacob T. Wilson, Anindita Banerjee, Karen A. Beningo

**Affiliations:** ^1^Laboratory of Cellular and Molecular Biology, Center for Cancer Research, National Cancer Institute, NIH, Bethesda, MD, United States; ^2^Department of Biological Science, Wayne State University, Detroit, MI, United States

**Keywords:** mechanotransduction, invasion, PAK, integrin beta-3, invadopodia

## Abstract

Cancer cells are affected by a wide range of mechanical forces within their extracellular environment. It has been widely shown that these forces can lead to increased metastatic activity of these cells. One such force is a transient tugging-like force that results from contractile forces generated by cells within the tumor microenvironment. When this force is simulated *in vitro* with a mechano-invasion assay, human fibrosarcoma cells exhibit enhanced cell invasion in a 3D collagen-fibronectin matrix by downregulating the expression of integrin β3. Furthermore, this force stimulates the maturation of invadopodia in an integrin β3-dependent manner that includes an increase in the active form of cofilin and MMP-2 secretion. In the present study we discovered that the decrease in integrin β3 signaling in response to mechanical stimulation is coupled to the activity of p21-activated kinase 1 (PAK1). It was found that PAK1 has decreased activity, as detected by a decrease in Ser144 phosphorylation, with mechanical stimulation. However, this loss in phosphorylation can be reversed if integrin β3 is overexpressed. Furthermore, PAK1 mutants show a correlated response in MMP-2 enzyme expression and activity, in addition to the lengthening of invadopodia, in response to stimulation. These results identify a novel mechano-sensitive response in human fibrosarcoma that utilizes PAK1 as a signaling player positioned downstream of integrin β3.

## Introduction

Cancer cells within a tumor are subjected to a wide array of biomechanical forces that can affect their metastatic capacity. Although the biochemical factors that influence metastasis have been well-studied, the mechanisms by which mechanical forces modulate the invasive capacity of cancer cells have received considerably less attention. Cells receive, interpret and respond to these forces via a process known as mechanotransduction, which is when an external mechanical signal is converted into an intracellular biochemical signal (Martino et al., 2018). Examples of biomechanical forces can include the stiffness of the extracellular matrix (ECM), interstitial flow and transient tugging forces (Joyce and Pollard, 2009; Kostic et al., 2009; Menon and Beningo, 2011; Kourouklis et al., 2016). Highly contractile cells that are located near a tumor, such as myofibroblasts, can produce a transient tugging force that is transmitted locally to nearby cells as they remodel the ECM (Wrobel et al., 2002). This type of transient force has been shown to increase the cell invasion of human fibrosarcoma cells within a 3D *in vitro* mechano-invasion assay. Increased cell invasion is dependent on the presence of fibronectin, an abundant ECM protein, and the expression of cofilin, an actin polymerization factor (Menon and Beningo, 2011). The transient tugging force causes decreased expression of the integrin β3 mechanoreceptor, which in turn causes protrusive structures called invadopodia to lengthen and produce more ECM-degrading matrix metalloprotease 2 (MMP-2) (Gasparski et al., 2017). Due to the increase in MMP-2 secretion and activity, fibrosarcoma cells increase their invasive capacity as they progress through the metastatic cascade. At present, the exact signaling mechanism that links mechanical force to the downregulation of the integrin β3 receptor and subsequently to increased cofilin activity is uncertain.

There is already an existing signaling pathway that links integrin β3 to the regulation of cofilin activity, but its role in this mechanosensitive process is unknown. Integrin β3 signals to Rac1 leading to the activation of p21-activated kinase 1 (PAK1) at the membrane by PAK1 autophosphorylation (Morgan et al., 2009). PAK1 then phosphorylates LIM kinase 1 (LIMK1) at the Tyr507 position, which reduces cofilin activity by phosphorylating cofilin at the Ser3 residue (del Pozo et al., 2000; Pollard and Borisy, 2003). However, it is not known if this pathway is relevant to the finding that transient mechanical stimulation produces an increase in cofilin activity, and as a result, increased invasion via the maturation of invadopodia. It is likely that this pathway could be downregulated, as this would produce more active, unphosphorylated cofilin leading to the maturation of invadopodia.

p21-activated kinase 1 is part of the six-member PAK serine-threonine-protein kinase family. It contains three main domains: a kinase domain in the C-terminal region, an auto-inhibitory domain and a p21-binding domain (Kumar et al., 2017; Rane and Minden, 2018). The auto-inhibitory domain within a single PAK inhibits the catalytic activity of its own kinase domain. A single PAK1 molecule is inactive but becomes active when its auto-inhibitory domain binds to another molecule’s kinase domain (Kumar et al., 2017). PAK1 is known to regulate the remodeling of the cytoskeleton, cell motility and invasion, metastasis and angiogenesis (Hammer et al., 2013; Hammer and Diakonova, 2015; Kumar and Li, 2016). The PAK family is an important link between the Rho family of GTPases and various cytoskeletal processes. For example, Rac1 is known to affect PAK1 signaling in both cell motility and invasion (Meyer Zum Buschenfelde et al., 2018). There is also significant evidence that PAK1 is involved in many types of cancer, especially in the regulation of metastatic capacity of invasive cells (Hammer et al., 2013; Hammer and Diakonova, 2015; Yang et al., 2015; Kumar and Li, 2016). However, to our knowledge, the present study is the first to examine the role of PAK1 in the response to this type of transient tugging force.

Through the use of an *in vitro* mechano-invasion assay that has been previously reported, we examined the role of PAK1 in the upregulation of invasion in response to transient mechanical stimulation. We found that PAK1 has both decreased expression and reduced phosphorylation (as shown by phospho-Ser144 levels) when transient stimulation is applied to human fibrosarcoma cells. When integrin β3 is overexpressed, phospho-PAK1 (p-Ser144-PAK1) levels are higher in stimulated cells, suggesting that PAK1 is more active. When mutants of PAK1 were expressed in these cells, the “kinase dead” mutants exhibited increased cell invasion, invadopodia maturation and corresponding MMP-2 secretion. Conversely, constitutively active PAK1 mutants showed less invasion, shorter invadopodia and less MMP-2 activity. These results suggest that a decrease in PAK1 activity is necessary for fibrosarcoma cells to enhance their invasiveness in response to mechanical stimulation. Elucidating the signaling pathway that is affected by mechanical stimulation within the tumor microenvironment can lead to a greater understanding of how cells can gain invasive capacity and progress through the metastatic cascade.

## Materials and Methods

### Cell Culture

Human HT1080 fibrosarcoma cells obtained from ATCC were cultured in Eagle’s Minimum Essential Media (EMEM; ATCC) supplemented with 10% fetal bovine serum (Hyclone) and 1% penicillin-streptomycin solution (100 U/ml penicillin and 100 μg/ml streptomycin; Life Technologies) at 37°C and 5% CO_2_ in a standard cell culture incubator. Cells were passaged via trypsinization with 0.25% trypsin (Sigma) and maintained up to the eighth consecutive passage. Cells were authenticated and tested negative for mycoplasma by the Biobanking and Correlative Services Core at the Karmanos Cancer Institute (Detroit, MI, United States).

### Integrin β3 Overexpression and PAK1 Mutants

For experiments requiring the overexpression of integrin β3, a plasmid containing the human *ITGB3* gene (pcDNA3.1-beta-3) from Timothy Springer (Addgene #272289) was used in HT1080 cells. For PAK1 mutant experiments, three plasmids were used: empty vector, PAK1-K299R (kinase inactive) and PAK1-T423E (constitutively active). These plasmids were a generous gift from Dr. Raymond Mattingly from the Department of Pharmacology at the Wayne State University School of Medicine (Detroit, MI, United States).

For all experiments using the above-mentioned plasmids, cells were allowed to grow to ∼80% confluency and transiently transfected via nucleofection using the Amaxa Nucleofector 2 device (Lonza) with kit T (Lonza).

### Mechano-Invasion Assay

The *in vitro* mechano-invasion assay was setup as described previously (Menon and Beningo, 2011). Briefly, a sterilized matrix containing collagen, fibronectin and paramagnetic beads was polymerized into a culture dish containing a well. Approximately 1.5 × 10^4^ HT1080 cells were seeded on top of the matrix. The entire assay was placed above a rotating rare earth magnet inside of a cell culture incubator for a period of 24 h. For unstimulated controls, the plate was incubated outside the magnetic field.

### Collagen Gel Degradation

The invasion assay matrix was degraded as previously described to collect the cells embedded on top of or invaded within the substrate (Menon and Beningo, 2011). Briefly, a 2 mg/ml solution of collagenase type IV (Worthington Biochemical) was prepared in Hank’s Balanced Salt Solution (Life Technologies) and warmed to 37°C. The matrix was physically removed from the culture dish and placed into the collagenase solution. With intermittent shaking, the matrix was allowed to degrade over a period of 10 min within a 37°C water bath. Cells were separated from the dissolved collagen by centrifugation and the pellet was washed once with sterile, warmed 1x phosphate buffered saline (PBS).

### Protein Extraction

Proteins were extracted from the collected cells as previously described (Menon and Beningo, 2011). Briefly, triple-detergent lysis buffer (TDLB; 50 mM Tris–HCl pH 8.0, 150 mM NaCl, 1% NP-40, 0.5% sodium deoxycholate, and 0.1% SDS) was mixed with Protease Inhibitor Cocktail (Sigma) and Halt^TM^ Phosphatase Inhibitor Cocktail (Thermo Fisher Scientific). The cell pellet was incubated with the lysis buffer solution for 20 min under ice-cold conditions. The solution was then centrifuged at 4°C for 10 min to isolate protein from cell debris. Protein concentration was determined by the DC protein assay (Bio-Rad).

### Conditioned Medium Collection

To collect secreted MMP-2 enzyme, the HT1080 cells were allowed to attach as normal to the invasion assay as described above. After the cells were attached, the medium was removed, and the matrix was rinsed twice with warmed 1x PBS. The medium was then replaced with serum-free medium for the duration of mechanical stimulation. The medium was collected by pipetting immediately before the matrix was degraded as described above. The collected medium was concentrated using 10 kDa molecular weight cut off protein concentrators (Thermo Fisher Scientific). Protein concentration was determined by the DC protein assay (Bio-Rad).

### SDS-PAGE and Western Blotting

Equal amounts of protein were mixed with 6x Laemmli buffer (Boston BioProducts) and boiled for 10 min (except for conditioned media for zymography, which was not boiled to retain enzyme activity). Samples were loaded onto an SDS-PAGE gel and allowed to separate. Proteins were transferred onto a PVDF membrane (Thermo Fisher Scientific) and blocked for 1 h at room temperature using 5% milk in 0.1% Tween-80 in PBS (PBS/T; for GAPDH), 5% milk in 0.1% Tween-80 in TBS (TBS/T; for integrin β3, MMP-2 and PAK1) or 5% bovine serum albumin (BSA; Santa Cruz) in 0.1% Tween-80 (BSA-TBS/T; for p-PAK1). Primary antibody dilutions were made in the same solution used for blocking and incubated overnight at 4°C. The following antibodies were used: rabbit polyclonal total PAK1 (1:1000; Bethyl Laboratories), rabbit monoclonal Ser-144 p-PAK1 (1:1000; Cell Signaling Technologies), rabbit polyclonal integrin β3 (1:300; Santa Cruz), mouse monoclonal GAPDH (1:7000; Millipore), HRP tagged anti-mouse (1:8000; Thermo Fisher Scientific) and HRP tagged anti-rabbit (1:10000; Amersham). The membranes were washed 3x for 10 min each in the same solutions used for blocking, but without milk or BSA added. Secondary antibody dilutions were made in the same solution used for the primary antibody and were incubated for 1 h at room temperature. After washing the membranes three times, the membranes were incubated with FemtoGlow WesternPLUS ECL reagent (Michigan Diagnostics). Band intensity readings were measured and normalized using ImageJ (NIH).

### Gelatin Zymography

Zymography was performed as described previously (Gasparski et al., 2017). Briefly, concentrated conditioned medium was mixed with 6x Laemmli non-reducing buffer (Boston BioProducts) and loaded onto an 8% SDS-PAGE gel containing 1 mg/ml gelatin (Sigma). After separation, the gel was incubated for 30 min at room temperature in renaturing solution (2.5% Triton X-100). The gel was then rinsed twice in water and incubated for 1 h at room temperature in developing buffer (50 mM Tris–HCl, pH 7.8, 0.2 M NaCl, 5 mM CaCl_2_, and 0.2% Triton X-100). The buffer was replaced with fresh developing buffer and incubated overnight at 37°C. The gel was rinsed with water and stained using 0.05% Coomassie blue and destained with methanol and acetic acid solution. Band intensities were measured using ImageJ (NIH).

### Immunofluorescence

Cells attached to the top and invaded within the invasion assay matrices were chemically fixed in a solution of 2.5% paraformaldehyde (Electron Microscopy Sciences) in 37°C 1.33x PBS for 10 min followed by a warmed solution of 2.5% paraformaldehyde, 1.33x PBS and 0.02% Triton X-100 for 10 min. Quenching was performed with a 0.5 mg/ml solution of NaBH_4_ (Sigma) in 1x PBS for 5 min at room temperature. Blocking was performed using 5% BSA (Santa Cruz) in 1x PBS overnight at 4°C. A mouse monoclonal anti-cortactin (1:500, cat# ab33333, Abcam) antibody was prepared in blocking solution overnight at 4°C. Samples were then incubated with secondary anti-mouse-IgG conjugated to Alexa Fluor 647 (1:450, Life Technologies) and Alexa Fluor 546 phalloidin (1:200, Life Technologies) prepared in blocking solution at room temperature for 1 h and followed by 3 washes in 1x PBS.

Matrices were imaged using a Leica SP8 confocal microscope with a 63x oil-immersion objective maintained by the Microscopy, Imaging and Cytometry Resources Core at Wayne State University (Detroit, MI, United States). Images were taken as z-stacks at 0.3 μm along the *z*-axis. Image stacks were analyzed and individual invadopodia measured using LAS X software (Leica) and as previously described (Gasparski et al., 2017).

## Results

### PAK1 Expression and Phosphorylation Levels Decrease With Mechanical Stimulation

**FIGURE 1:**
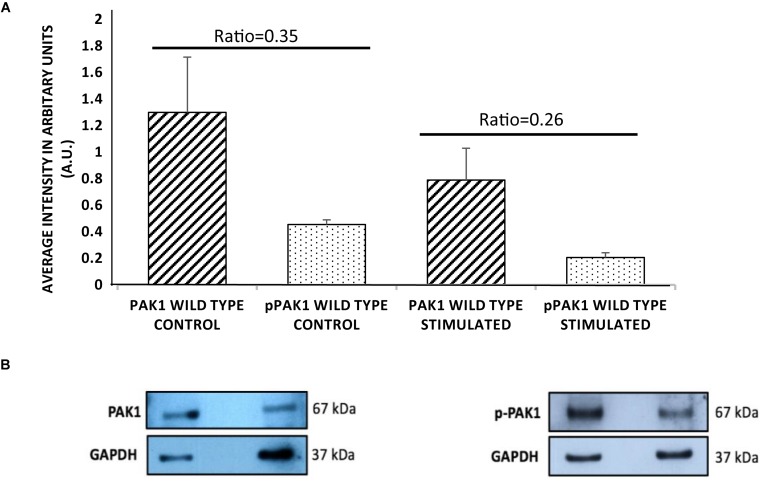
p21-activated kinase 1 expression and phosphorylation decrease with mechanical stimulation. **(A)** HT1080 cells show significantly decreased total PAK1 levels with stimulation compared to unstimulated cells. In addition, Serine 144 phospho-PAK1 levels significantly decrease with mechanical stimulation, suggesting that PAK1 activity is also decreased with stimulation. **(B)** Representative western blots of PAK1 and pPAK1 using GAPDH as load controls. The difference in ratio between stimulated and unstimulated cultures was highly significant, *P* < 0.01 by Student’s *t*-test. Errors bars show the SEM, *n* = 3.

In order to determine if PAK1 is involved in the response to mechanical stimulation in human fibrosarcoma cells, it was necessary to examine the total expression level and the level of active PAK1 protein. PAK1’s activity is regulated by phosphorylation at the serine 144 position by Rac1-GTPase, so we also examined Ser144 p-PAK1 with and without stimulation. When mechanical stimulation is provided by the mechano-invasion assay, the expression of total PAK1 is reduced by approximately 60% compared to unstimulated cells (Figure 1). Similarly, the level of Ser144 p-PAK1 is also reduced by ∼46% with transient tugging mechanical force. The ratio of pPAK/PAK in unstimulated cells is 0.35 and 0.26 in stimulated cells. These results suggest that mechanical stimulation reduces both the expression of PAK1 and the level of active PAK1 as indicated by phosphorylation of Ser144.

### Overexpression of Integrin β3 Increases PAK1 Phosphorylation Levels

**FIGURE 2:**
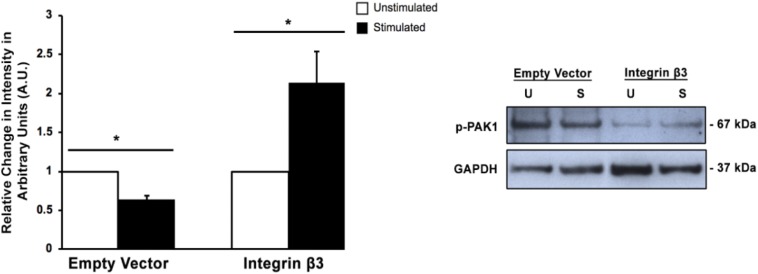
Overexpression of integrin β3 affects PAK1phosphorylation levels. When integrin β3 is overexpressed in HT1080 cells, mechanical stimulation produces an increase in p-PAK1 levels, which suggests that integrin β3 signaling is required for the activation of PAK1. The empty vector represents cells that have been mock transfected using GFP plasmid. ^∗^*P* < 0.05 by the Student’s *t*-test; *n* = 3. Error bars represent the SEM.

Since we had previously shown that the expression of integrin β3 mechanoreceptor is downregulated when transient mechanical stimulation is applied to HT1080 cells (Gasparski et al., 2017), it seemed prudent to examine if affecting integrin β3 levels would change the phosphorylation of PAK1 and subsequent activity. Confirmation of this result would suggest that PAK1 is involved in the intracellular signaling cascade that is affected by the loss of integrin β3 expression. To do this, we transiently overexpressed the human integrin β3 gene (*ITGB3*) in HT1080 cells and examined Ser144 PAK1 phosphorylation levels with and without stimulation. We found that when integrin β3 is overexpressed, the level of p-PAK1 increases with stimulation, which suggests PAK1 is more active when being stimulated by integrin β3 and its downstream effectors in the signaling axis (Figure 2). This is compared to the empty vector control where mechanical stimulation continued to produce a decrease in p-PAK1 levels. These results confirm that the regulation of PAK1 activity is downstream of integrin β3 signaling in response to stimulation.

### Enhanced Cell Invasion in Response to Stimulation Depends on Reduced PAK1 Phosphorylation

**FIGURE 3:**
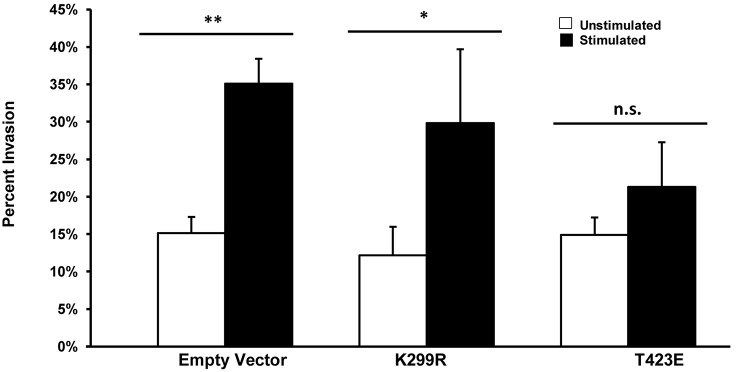
Enhanced invasion in response to stimulation requires lower phosphorylation levels of PAK. In cells with PAK1 activity reduced (K299R), mechanical stimulation results in an enhanced level of cell invasion. When PAK1 activity is increased (T423E), stimulation no longer leads to more cell invasion. ^∗^*P* < 0.05, ^∗∗^*P* < 0.01 by Student’s *t*-test, data from at least three independent experiments. Error bars represent SEM ± mean.

We have previously shown that transient mechanical stimulation leads to enhanced cell invasion (Menon and Beningo, 2011), but the signaling pathway that is involved in this response has not been elucidated. To determine if PAK1 is a member of the signaling pathway that produces enhanced invasion in response to stimulation, we modified the activity of PAK1 in cells through the use of two PAK1 mutants. To decrease PAK1 activity, we transiently overexpressed a plasmid containing the PAK1 gene possessing a K299R substitution (PAK1-K299R), considered kinase dead, in HT1080 cells and tested their invasion in our mechano-invasion assay. After stimulation, cells containing the kinase dead PAK1 exhibited increased invasion in response to stimulation, similar to the cells expressing the negative control vector (Figure 3). To examine when PAK1 activity is constitutively active, a plasmid containing a T423E substitution in the PAK1 gene (PAK1-T423E) was nucleofected into the cells and mechanical stimulation was applied via our assay. Upon doing so, the enhancement of invasion was lost, which suggests that a decrease in PAK1 activity is necessary to promote enhanced invasion in response to transient mechanical stimulation.

### Invadopodia Become Shorter in Length With Modified PAK1 Activity

**FIGURE 4:**
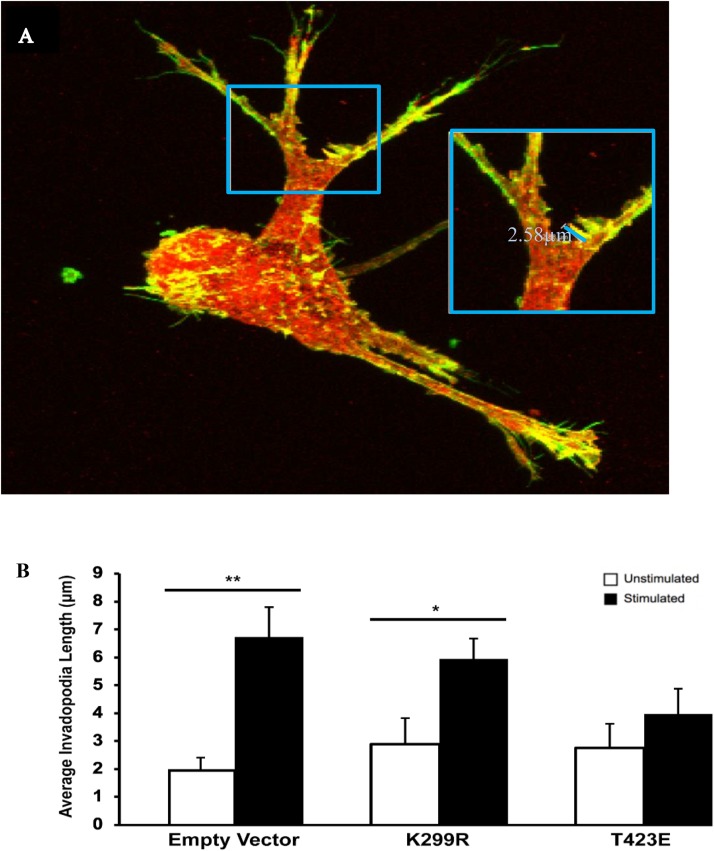
**(A)** The length of invadopodia can be measured based on actin and cortactin colocalization represented as yellow, images collected by confocal microscopy. **(B)** Invadopodia fail to elongate in response to transient mechanical stimulation with an overactive PAK1. When PAK1 is made less active in HT1080 cells (K299R), invadopodia continue to elongate in response to transient mechanical stimulation. However, when PAK1 is constitutively active (T423E), invadopodia do not elongate in response to stimulation. ^∗^*P* < 0.05, ^∗∗^*P* < 0.01 by Student’s *t*-test, data from at least three independent experiments. Error bars represent the SEM.

In a previous study, we found that transient mechanical stimulation promotes the elongation of invadopodia through the downregulation of the integrin β3 receptor (Gasparski et al., 2017). However, the exact signaling mechanism for this is not clear. To determine if PAK1 is involved in this pathway, we increased and decreased its activity level and measured invadopodia with and without stimulation. To modify PAK1 activity, we expressed the PAK1 mutant vectors in HT1080 cells and plated them on our mechano-invasion assay. The cells in the collagen-fibronectin matrix were then fixed within the matrix, stained with fluorescent antibodies for cortactin and actin (invadopodia markers) and imaged using z-stack confocal microscopy. When PAK1 activity is reduced in cells subjected to mechanical stimulation (PAK1-K229R), invadopodia elongate in length similar to stimulated cells containing the control empty vector (Figure 4). Furthermore, when PAK1 activity is made constitutively active via the PAK1-T423E plasmid, invadopodia did not exhibit any change in length between unstimulated and stimulated cells. This suggests that having less PAK1 activity is necessary for mechanical stimulation to promote the lengthening of invadopodia in fibrosarcoma.

### Overexpression of Phosphorylation Defective PAK1 Increases MMP-2 Expression and Secretion

To confirm that PAK1 activity is affecting the maturation of invadopodia, we used the same three PAK1 mutant plasmids to examine the expression and secretion of MMP-2, an invadopodia-associated enzyme. MMP-2 is a matrix metalloprotease that has been shown to be secreted by fully mature invadopodia (Jacob and Prekeris, 2015). Its function is to degrade the surrounding ECM components, primarily collagen, to facilitate easier cell invasion. We have previously shown that transient mechanical stimulation increases both MMP-2 expression and secretion (Gasparski et al., 2017). As a result, a corresponding change in MMP-2 activity when PAK1 is either more or less active would be expected.

**FIGURE 5:**
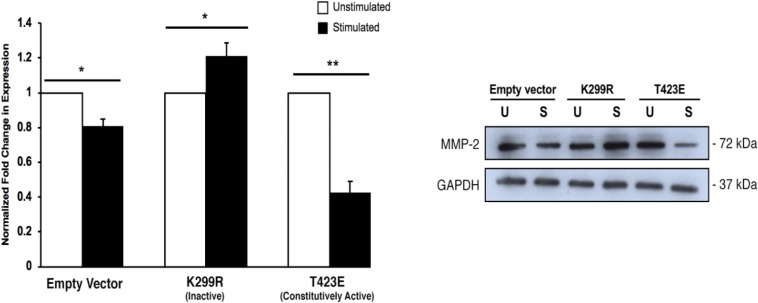
Intracellular MMP-2 expression increases with when the PAK1 kinase-dead mutant is overexpressed. When PAK1 is inactive (K299R), there is a significant increase in intracellular MMP-2 expression with stimulation. When PAK1 is constitutively active (T423E), there is a decrease in MMP-2 expression with stimulation. ^∗^*P* < 0.05, ^∗∗^*P* < 0.01 by Student’s *t*-test, *n* = 3. Error bars represent the SEM.

When PAK1 is made inactive via expression of the PAK1-K299R plasmid, we found that there is an increase in intracellular MMP-2 expression with stimulation. Conversely, when PAK1-T423E is expressed in cells, mechanical stimulation significantly decreases (∼60%) the expression of MMP-2 (Figure 5). This result suggests that more MMP-2 is being intracellularly expressed when PAK1 is less active.

**FIGURE 6:**
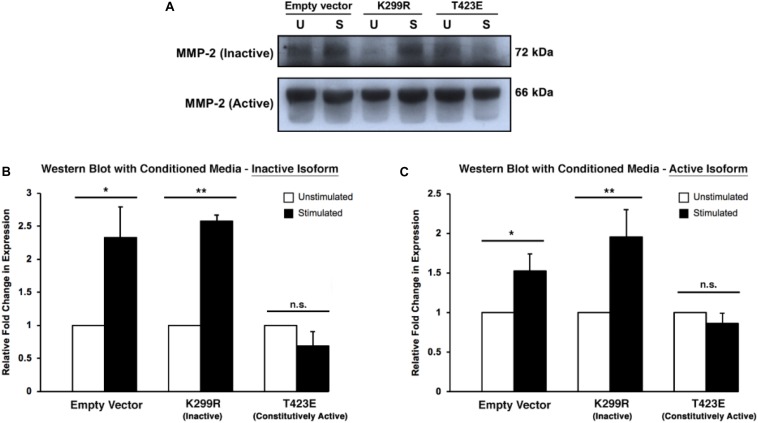
Overexpression of kinase-dead PAK1 promotes more MMP-2 secretion in response to mechanical stimulation. **(A)** A representative western blot of PAK1 mutants (K299R: kinase inactive; T423E: constitutively active) and inactive/active MMP-2 isoforms. **(B)** When PAK1 is made inactive, there is more inactive MMP-2 secretion with stimulation compared to overactive PAK1. **(C)** With stimulation, a less active PAK1 causes a significant increase in MMP-2 section of the active isoform. If PAK1 is constitutively active, no change in MMP-2 secretion occurs with stimulation. ^∗^*P* < 0.05, ^∗∗^*P* < 0.01 by Student’s *t*-test; *n* = 3. Error bars represent the SEM.

Because MMP-2 is a secreted protease, it is important to measure its level within the culture medium. To do this, conditioned medium was collected from the invasion assay plates just prior to collagen degradation and protein extraction. The medium was concentrated using a protein concentration column to remove proteins that are vastly different sizes than MMP-2. We found that kinase inactive (PAK1-K299R) cells had increased levels of both inactive and active isoforms of MMP-2 within the media (Figure 6). Furthermore, cells expressing the constitutively active PAK1 (PAK1-T423E) had no significant change in MMP-2 secretion with mechanical stimulation. These results suggest that having less active PAK1 causes more MMP-2 to be secreted from more mature invadopodia.

These results further confirm that changing PAK1 activity in fibrosarcoma cells affects invadopodia maturation in response to mechanical stimulation. Because MMP-2 expression is a hallmark of mature invadopodia, there should be an increase in both expression and secretion of MMP-2 if invadopodia are indeed becoming more mature in response to mechanical stimulation.

### Overexpression of “Kinase-Dead” Mutant PAK1 Promotes MMP-2 Degradative Activity

**FIGURE 7:**
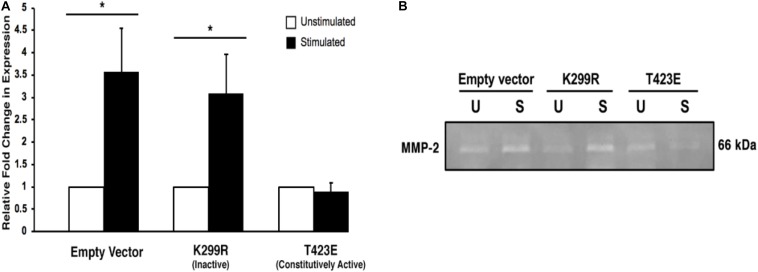
Overexpression of the kinase-dead mutatnt PAK1leads to more active MMP-2-mediated degradation. **(A)** When PAK1 (K299R) is transiently overexpressed, there is a significantly greater gelatin degradation with mechanical stimulation. However, when the constitutively active PAK1 is overexpressed mechanical stimulation does not affect MMP-2’s enzymatic activity. ^∗^*P* < 0.05 by Student’s *t*-test, *n* = 3. Error bars represent the SEM. **(B)** A representative image of a gelatin zymography using conditioned medium contains the active form of MMP-2 from empty vector and PAK1 mutant cells.

It is important to measure the activity of the secreted MMP-2 enzyme, since only active form can lead to the increase in cell invasion through degradation of the surrounding ECM. We used gelatin zymography to determine the activity level of the MMP-2 within the collected conditioned medium. We found that with mechanical stimulation, transiently expressed kinase inactive PAK1 (PAK1-K299R) had significantly greater (∼2.5-fold) gelatin degradation activity compared to unstimulated cells (Figure 7). When PAK1 is transiently overexpressed as its constitutively active form (PAK1-T423E), no change is observed in the extent of gelatin degradation between mechanically stimulated and unstimulated cells. These data indicate that the elevated MMP-2 secreted from the kinase inactive PAK1 cells is enzymatically active. Since there is an increase in longer invadopodia in these cells, a corresponding increase in MMP-2 expression and activity suggests that these invadopodia are also more mature.

## Discussion

Metastasis is a multi-step process that requires a cell to invade and migrate through several varieties of environments. As an invading tumor cell moves through these different environments, it will encounter an array of mechanical forces. How the cell reacts and responds to these forces can affect its ability to progress through the metastatic cascade. Therefore, it is important to study these forces and the cellular mechanisms involved in responding to them. The force investigated in this study is a transient tugging force that is produced by nearby cells migrating through and remodeling the ECM near a tumor. Using a previously developed *in vitro* mechano-invasion assay (Menon and Beningo, 2011), we mimicked these transient tugging forces on a collagen-fibronectin matrix and examined the cell’s invasive physiology. This force is one that has been shown to enhance the invasion of highly invasive cancer cells by increasing the maturation of invadopodia (Gasparski et al., 2017). However, the signaling mechanism that is involved in this process is not yet clear.

**FIGURE 8:**
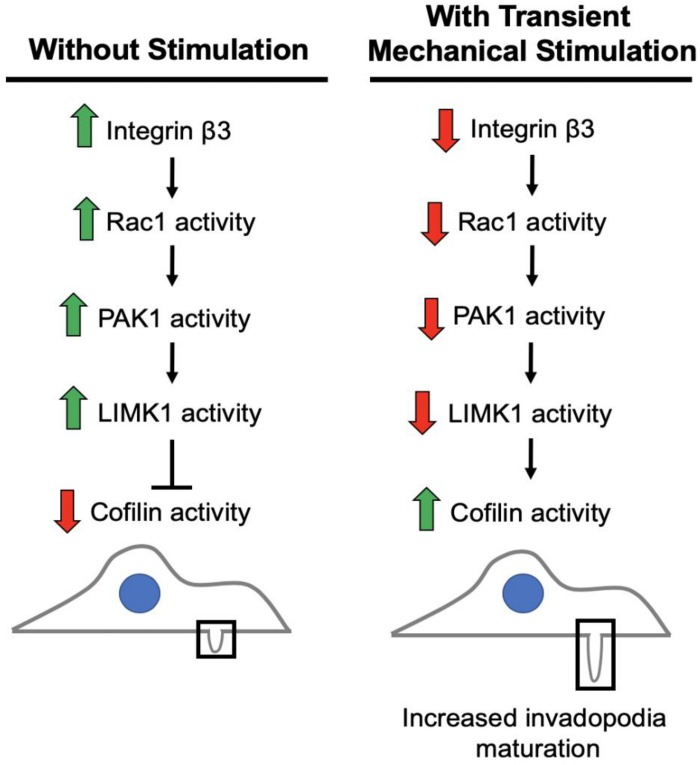
Working model of the pathway to maturation of invadopodia when cancer cells are stimulated by transient tugging forces. The above signaling pathway is proposed to lead to the enhanced invasion observed in fibrosarcoma cells when transient tugging forces are applied through a three-dimensional mechano-invasion assay. Invadopodia elongate and secrete more MMP-2 when the protein expression of integrin beta-3 is downregulated in response to tugging forces. Cofilin is most active when it is not phosphorylated and serves as a kinase substrate of LIMK1, which is activated by PAK1 and subsequently Rac1. Rac1 and LIMK1 have not yet been confirmed to participate in this mechanosensitive pathway.

We previously found that integrin β3, and not integrin β1, as important in this process as its downregulation is necessary to enhance invasion in response to transient stimulation (Gasparski et al., 2017). Based on our studies and current literature, we believe integrin β3 to be connected to the regulation of cofilin activity via a PAK1 signaling axis (Figure 8). We investigated whether this PAK1 signaling pathway is utilized by cells to upregulate their invasion in response to mechanical stimulation. Using our mechano-invasion assay we tested PAK1 expression and phosphorylation with mechanical stimulation, in addition to measuring invadopodia and MMP-2 levels.

We found that transient mechanical stimulation caused a decrease in PAK1 expression and phosphorylation level, as measured by Ser144 phosphorylation. Also, when integrin β3 is overexpressed, the decrease in PAK1 expression and phosphorylation is abolished, suggesting that PAK1 is downstream of integrin β3. To determine if invadopodia maturation is also regulated by the phosphorylation state of PAK1, we transiently expressed PAK1 mutants (kinase inactive and constitutively active) in cells and measured the length of invadopodia. When PAK1 is less phosphorylated, invadopodia still become longer in response to mechanical stimulation. However, when PAK1 is constitutively active, invadopodia do not lengthen with stimulation, which suggests that a decrease in PAK1 activity is required for the cell to produce longer invadopodia to upregulate their invasion in response to tugging forces.

Increased matrix metalloprotease activity is a hallmark of mature invadopodia. We tested the levels of MMP-2, an invadopodia-associated protease, in PAK1 mutant cells (Jacob and Prekeris, 2015). When PAK1 phosphorylation is decreased, more MMP-2 is secreted and enzymatically active compared to when PAK1 is constitutively active through transient overexpression. This again suggests that a decrease in PAK1 activity is important in producing mature, enzymatically active invadopodia.

There are other components of the signaling pathway that must be involved, as PAK1 is not known to directly interact with cofilin and we are currently pursuing the working model illustrated in Figure 8. The kinase that is responsible for phosphorylating cofilin at the Ser3 position to regulate its activity is LIM kinase 1 (LIMK1) (Pollard and Borisy, 2003). PAK1 is a known regulator of LIMK1 (Park and Kim, 2017), so it is reasonable to hypothesize that a decrease in PAK1 activity produces less active LIMK1, which would mean less Ser3-phosphorylated, inactive cofilin. With cofilin being more active, it would be able to promote further actin polymerization at nascent invadopodia resulting in elongation and maturation in response to this type of mechanical stimulation. Further data is needed to confirm that LIMK1 is part of this integrin β3-Rac1-PAK1 signaling axis. It is also possible that PAK1 might be affecting another aspect of invadopodia maturation, such as modifying the activity of another molecule involved in promoting their maturation. Additionally, this process should be explored in other cancer types beyond fibrosarcoma, as there are often cell type-dependent differences.

Overall, this study demonstrates the importance of examining the impact of mechanical forces within the tumor microenvironment. It is becoming increasingly evident that these mechanical forces can cause cancer cells to become more invasive and thus, more metastatic. Understanding the mechanotransduction signaling pathways affected by these forces can lead to therapeutic targets for future treatments to reduce cancer cell metastasis.

## Data Availability Statement

The datasets generated for this study are available on request to the corresponding author.

## Author Contributions

AG and JW performed the experiments and writing. AB contributed to the experiments and editing. KB provided funds and materials, and contributed to the conceptualization and editing.

## Conflict of Interest

The authors declare that the research was conducted in the absence of any commercial or financial relationships that could be construed as a potential conflict of interest.
